# Who Is Running in the D-A-CH Countries? An Epidemiological Approach of 2455 Omnivorous, Vegetarian, and Vegan Recreational Runners—Results from the NURMI Study (Step 1)

**DOI:** 10.3390/nu14030677

**Published:** 2022-02-05

**Authors:** Katharina Wirnitzer, Mohamad Motevalli, Derrick Tanous, Gerold Wirnitzer, Claus Leitzmann, Renato Pichler, Thomas Rosemann, Beat Knechtle

**Affiliations:** 1 Department of Research and Development in Teacher Education, University College of Teacher Education, Tyrol, 6010 Innsbruck, Austria; seyed.motevalli-anbarani@student.uibk.ac.at (M.M.); Derrick.Tanous@student.uibk.ac.at (D.T.); 2 Department of Sport Science, University of Innsbruck, 6020 Innsbruck, Austria; 3 Research Center Medical Humanities, Leopold-Franzens University of Innsbruck, 6020 Innsbruck, Austria; 4 AdventureV & Change2V, 6135 Stans, Austria; gerold@wirnitzer.at; 5 Institute of Nutrition, University of Gießen, 35390 Gießen, Germany; claus@leitzmann-giessen.de; 6 Niederfeldstrasse 92, 8408 Winterthur, Switzerland; renato.pichler@dietethics.eu; 7 Institute of Primary Care, University of Zurich, 8000 Zurich, Switzerland; thomas.rosemann@usz.ch (T.R.); beat.knechtle@hispeed.ch (B.K.); 8 Medbase St. Gallen Am Vadianplatz, 9000 St. Gallen, Switzerland

**Keywords:** prevalence, diet, plant-based, athletes, performance, endurance running, training, racing, half-marathon, marathon

## Abstract

Accompanied by the growing popularity of distance running, the prevalence of vegan and vegetarian diets in endurance runners has increased across the globe and especially in German-speaking (D-A-CH: Germany, Austria, Switzerland) countries. The present study aimed to investigate and compare the epidemiological characteristics associated with diet types and running behaviors of recreational endurance runners. From a total number of 7422 runners who started to fill in the online survey, 3835 runners completed the questionnaire. After data clearance, 2455 distance runners (mean age: 37 years; 56% females, 44% males) were selected as the final sample and classified as 1162 omnivores (47.4%), 529 vegetarians (21.5%), and 764 vegans (31.1%). Sociodemographic information and general characteristics in training and competition were evaluated using a questionnaire-based approach. A significant association was found between diet type and race distance (*p* < 0.001). In females, vegan ultra-marathoners and omnivorous half-marathoners had better individual running records among dietary groups. Sex differences in running performance had a minimizing trend with increasing race distance. Most runners reported independent race preparation (90%) over less than four months (73%). From an epidemiological viewpoint, the present findings suggest a central role of plant-based diets in running performance and behaviors among active distance runners in D-A-CH countries and that vegetarian and vegan diets are compatible with competitive running.

## 1. Introduction

As one of the most popular physical activities, recreational running is an affordable and influential health-promoting approach at all ages [1]. This low-intensity, high-duration activity is performed over various distances but mainly from 5 km to ultra-marathon, including 100-km runs [2]. During the past decade, a 60% increase in the popularity of endurance running has been reported, and currently, over 70,000 endurance running events are held annually worldwide [2]. It has been reported that the major motives to participate in distance running events include personal achievement, health promotion, weight control, pleasure, well-being, winning, competitive opportunities, as well as social and communicational reasons [3,4]. However, as a result of the current COVID-19 pandemic, data indicate that runner motivation has partially shifted from competition and socialization towards fitness, recovery, and stress relief [5]. Nevertheless, runner motivation may vary based on race distance, as longer-distance runs (e.g., marathon and ultra-marathon) are known to be more demanding and may lead to multiple physical and physiological challenges [6,7]. 

Over 50 million Europeans have been reported as active runners at the recreational or professional level [8]. A recent study by *Asics* found that only 15% of European runners have completed a marathon, and 37% plan to run a marathon in the near future [9]. During the recent decades, it has been stated that runners from German-speaking countries (also called D-A-CH countries) make up a remarkable portion of marathon and ultra-marathon finishers [10,11]. This occurrence might be related to the fact that a considerable number of running events are held annually in D-A-CH countries, including the Berlin Marathon, which is considered among the top five largest marathon events worldwide [12].

Nutrition plays a remarkable role in running adaptations and racing performance [13], and diet type could be an important indicator of daily nutrient requirements and, consequently, successful endurance runs [14]. Vegan (defined as a diet devoid of all ingredients from animal sources) and vegetarian (defined as a diet devoid of meat and flesh foods) nutrition are two major categories of diet types that are followed for various reasons (including health, physical performance, ethical issues, and environmental concerns) [15,16]. Although some reports indicate a lower bioavailability of certain nutrients (e.g., protein, iron, zinc, vitamin D, vitamin B_12_) from plant foods [17,18], it has been well documented that a well-planned vegan diet can fulfill all nutritional requirements [15,17,19]. However, vegetarian/vegan diets naturally contain higher amounts of antioxidants, carbohydrates, and many micronutrients (e.g., vitamin C, folic acid, magnesium, potassium) [19,20,21], which may bring about beneficial significances in endurance running performance [15]. From a practical viewpoint, however, evidence indicates that if the well-recognized dietary requirements are addressed by a well-planned diet, a purely plant-based or vegan diet can fulfill the nutritional needs for the successful and healthy completion of all events, including ultra-endurance, multi-stage races [21,22]. 

According to Google, Forbes, and The Economist, the prevalence of vegetarian and vegan diets is growing globally at a rate faster than expected [21]. It has been reported that 10% of European populations (about 75 million) follow a vegan or vegetarian diet [23]. Findings from a recent European survey performed on a large sample of the general population from seven European countries [24] showed that the prevalence of vegan/vegetarian diet in D-A-CH countries is 2.1% higher than the European average. Reports from another study showed that 10–14% of people in D-A-CH countries follow a vegetarian or vegan diet [22,25,26]. A recent nutrition report found that the number of vegetarians and vegans in Germany has doubled since the onset of the COVID-19 pandemic [27].

As the interest in the vegan diet in sports for reasons of health and performance gains more and more attention in athletic populations, the significance of this vegan boom is embellished by the victories of elite-class vegan athletes at World Championships, World Series, Olympic Games, and other top-tier events, which is reflected by documentaries, such as “The Game Changers” [15,21]. Thus, while a large number of recreational and professional athletes follow vegetarian diets, the vegan diet has been established even amongst world-famous professional athletes, such as Lewis Hamilton (Formula 1), Novak Djokovic (tennis), Gerlinde Kaltenbrunner (mountaineering), Fiona Oakes (marathon/ultra-marathon), and Scott Jurek (ultra-marathon) [21]. Evidence shows that about 10% of marathoners follow vegan or vegetarian diets [28], with an increasing trend in higher distances (e.g., ultra-marathon) [29]. Despite the growing prevalence of plant-based diets, research on the athletic population is still challenging, as it needs to find and control a group of vegetarian/vegan athletes participating in the same sport to provide a sufficiently large sample for a meaningful study [14]. Results from a study investigating the association between diet types and running distances found that not only the prevalence of vegan/vegetarian diets in ultra-marathoners was higher, but they also reported following a vegetarian/vegan diet for a longer extent compared to half- and full-marathoners [29]. However, although no difference has been reported in diet quality scores between runners in different race distances, vegan and vegetarian runners were found to have higher diet quality scores than their omnivorous counterparts [29]. 

Regardless of nutrition, the endurance runner lifestyle is highly influenced by training activities and competition schedules. Several individual and running-associated variables (e.g., age, sex, motivation, environment, race distance, nutrition, preparation phases, competition level) have been shown to affect runners’ training patterns [30,31,32]. To improve running adaptations and performance, it is recommended to plan and conduct training with great precision over different loading phases (including high volume, high intensity, and workout diversity) [33]. Public beliefs indicate that preparation for an endurance event requires long endurance runs performed within the pre-competition days/weeks in order to reach a high workload [32,34]. An inappropriately increased training load, however, may not guarantee success and can potentially result in detrimental consequences to health (e.g., running-related injuries and chronic pain) [7,35] and performance [31]. The mean training volume of marathon runners has been shown on average to be about 50 km/week with a running speed of 11 km/h [34,36]. Evidence indicates that training volume seems to be associated with race distance, as the highest training kilometers among distance runners has been reported in ultra-marathoners, and contrariwise, half-marathoners have shown a lower weekly training mileage than marathoners [37,38]. However, race distance could greatly affect training volume, as it has been shown that half-marathoners focus more on training speed, while ultra-marathoners concentrate more on training duration [38]. In this regard, supervision from a specialist could result in beneficial advantages in training adaptations and running performance [39,40]. When comparing race distances, evidence shows that endurance runners competing in long distances (i.e., marathon and ultra-marathon) have a greater tendency to be supervised by a specialist (e.g., sports scientists, sport medicine physicians) [41]. In general, however, as scientific evidence investigating endurance runner behavior is mainly based on studies observing a mixed group of recreational and professional runners with a varied level of fitness and experience, caution is recommended when comparing training and racing behaviors of pooled groups of distance runners [30].

Despite the availability of a broad range of studies investigating different aspects of vegan/vegetarian diets or endurance performance [42,43,44], little is known about the prevalence of vegan and vegetarian diets and their connections with running behaviors among distance runners. Considering the rising number of vegan/vegetarian athletes, on the one hand, and the growing popularity of endurance running on the other hand [2,21], the present study aimed to assess the epidemiological characteristics of vegan, vegetarian, and omnivorous endurance runners as well as their running-associated behaviors in a large population of recreational runners from the D-A-CH countries. 

## 2. Materials and Methods

### 2.1. Study Protocol and Ethics Approval

The present study is a part of the Nutrition and Running High Mileage (NURMI) Study. The NURMI Study is considered the largest European study on runner populations (www.nurmi-study.com/en; accessed on 4 February 2022) and was conducted in three steps following a cross-sectional design. Step 1 (in which the present study belongs) aimed to determine the prevalence of vegan and non-vegan recreational athletes in running events by using a standardized questionnaire following an epidemiological approach. Detailed information about the methods of the Step 1 study has been described elsewhere [30,41]. The NURMI Study protocol [45] was approved by the ethics board of St. Gallen, Switzerland (May 6, 2015; EKSG 14/145). The trial registration number is ISRCTN73074080 (retrospectively registered 12 June 2015).

### 2.2. Participants

Male and female recreational runners were contacted and recruited mainly via social media, websites of the organizers of marathon events, online running communities, email lists and runner magazines, as well as via magazines for health, nutrition, and lifestyle; trade fairs on sports, plant-based nutrition, and lifestyle; and through personal contacts within runners’ communities. The regions intended to be mainly addressed were the German-speaking countries (i.e., D-A-CH countries), including Austria, Germany, and Switzerland. 

### 2.3. Experimental Approach 

Participants completed a short online survey within the NURMI Study Step 1 available in German and English on www.nurmi-study.com/en (01.10.2014–31.12.2015). The survey started with a written description of the procedure, and runners gave their informed consent to participate in the study. Subsequently, they completed the short and standardized questionnaire, which included questions concerning demographic characteristics, diet types (current diet; intake of specific foods, such as fruit, vegetables, grains, meat, fish, dairy products), and running characteristics of training sessions and races, including specified questions to ensure their involvement in at least one running event within the past two years. Two groups of control questions were included for measures of “diet” and “running activity” in order to increase the data validity within each different section of the questionnaire.

For complete participation in the study, the following five inclusion criteria were required/assessed: (1) written informed consent, (2) minimum age of 18 years, (3) completion of the questionnaire Step 1, (4) completion of at least one running event in the past two years, and (5) currently active in running. Those who met all inclusion criteria were included in the study. 

#### Participants’ Classifications

Participants were classified into three dietary subgroups: omnivorous (commonly known as “the Western diet,” with no dietary restrictions), vegetarian (no meat, fish, or meat products), and vegan (no products from animal sources, such as meat and processed meat, fish, seafood and shellfish, milk and dairy products, eggs, or honey) [46]. Analysis of control questions showed that a total of 107 runners (4% of the study sample) had to be shifted to other dietary subgroups: 5 vegan runners (4 to omnivores and one to vegetarians) and 102 vegetarian runners (all to omnivores). Moreover, 96% of the total runners (*n* = 2348) reported a correct assessment of their diet type. 

The participants were categorized into three subgroups regarding race distances: distances shorter than half-marathon (<21 KM; mainly 5 km, 8 km, and 10 km), half-marathon (HM), and marathon/ultra-marathon (M/UM). Data for the marathon and ultra-marathon runners were pooled since the marathon distance is usually included in different stages of an ultra-marathon event. The shortest ultra-marathon distance reported was 50 km, and the longest distance was 160 km. Highly motivated runners (*n* = 535) who had not successfully participated in either a HM, M, or UM but instead competed in races over distances shorter than HM (<21 KM) provided accurate and useful answers with plenty of high-quality data. Therefore, to avoid an irreversible loss of these valuable data sets, those who met all inclusion criteria but reported races shorter than half-marathon (<21 KM) were included as an additional race distance category.

### 2.4. Data Clearance 

A total number of 7422 runners started to fill in the online survey. However, 48% dropped out and did not complete the survey. As a result, a total of 3835 runners completed the survey comprising the study sample. Incomplete, inconsistent, or conflicting data sets were excluded from data analysis (*n* = 1242). Of those excluded, a total of 833 runners did not meet the inclusion criteria, and 409 were from non-D-A-CH countries. In addition, to ensure a minimal health status linked to a minimum fitness level and to further enhance the reliability of data sets, a BMI-related supplementary criterion was implemented following the WHO approach, which points to a moderate to severe risk of comorbidities in people with a BMI > 30 kg/m^2^ [47,48]. However, with a BMI ≥ 30, other health protective and/or weight loss strategies besides running are first necessary to safely reduce body weight. Since some people with a high BMI might have little sport-associated motives and may start running to achieve a healthy body weight, participants with a BMI ≥ 30 were excluded from the data analysis (*n* = 65). Furthermore, 95 runners were also excluded from the final sample due to inconsistent information about running races, particularly their best time in HM and M.

**Figure 1:**
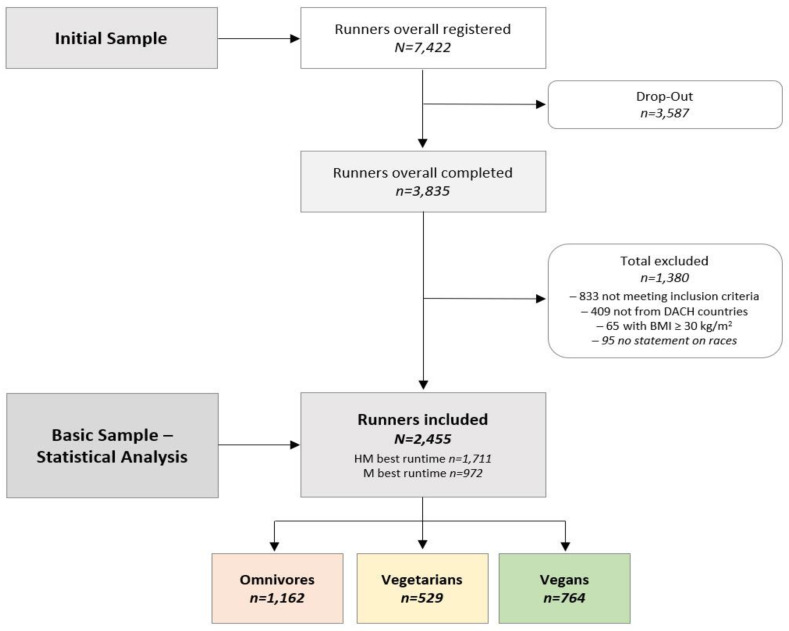
Flow of participants’ enrollment and dietary subgroups. BMI, body mass index; D-A-CH, Germany, Austria, and Switzerland; Omnivores, those who have no restriction on source of food; Vegetarians, those who avoid all flesh foods but consume egg and/or dairy products; Vegans, those who avoid all foods and ingredients from animal sources.

After data clearance, a total of 2455 runners with complete and correct data sets were included in the descriptive analysis. With a cross-reference to the whole NURMI Study (particularly Steps 2 and 3), the present investigation focused on active runners who are at least able to cope with the HM distance to ensure the assessment of fit runners only, and thus, 2455 recreational runners active in races over 10-km distance were included in the inferential statistical analysis. Figure 1 shows the flow of participants´ enrollment in the NURMI Study Step 1 and the classification based on their diet type.

### 2.5. Measures

An epidemiological approach was used to investigate the prevalence of omnivorous, vegetarian, and vegan recreational runners linked to their running characteristics and sociodemographic information, including nationality, age, sex, body weight, height, and BMI_CALC_ [47,48]. In addition, the prevalence of the diet type was performed by the following items: adherence to different kinds of diet (mixed, vegetarian, vegan); duration of adherence to an omnivorous, vegetarian, or vegan kind of diet; consumption of specific food items; and nutrient and fluid intake on race days. Moreover, the prevalence of (endurance) runners at the start of running events was described by the following items: training behavior (weekly/daily time spent in running/day); period of time to prepare for the main running event; aim of taking part in a running race (performance vs. pleasure approach); participation in running events, distance/s that have been completed (<21 km, HM, M, UM), number of completion of specific distance/s, and individual best time over HM and/or M distance/s.

### 2.6. Statistical Analysis

The statistical software R version 4.1.1 (2021-08-10), Core Team 2018 (R Foundation for Statistical Computing, Vienna, Austria) performed all statistical analyses. Exploratory analysis was performed by descriptive statistics (mean values and standard deviation (SD), median, and interquartile range (IQR)).

Significant differences between dietary subgroups and sex, age, BMI, race distance, and running/racing behaviors were calculated using a non-parametric ANOVA. Univariate analysis was performed with chi-square test (χ^2^; nominal scale), and Kruskal–Wallis test (ordinal and metric scale) examined the association between variables approximated by using the *t* or F distributions with ordinary least squares and standard errors (SE) and R^2^. A linear regression model was used to examine the performance (best time over the HM and M distance). The regression model included diet and sex, age, and BMI as potential confounders. The assumptions of the regression analysis were verified by inspection of graphs of residuals. Effect plots (95% confidence interval (95%CI)) display the differences in dietary subgroups by race distance and performance in female and male runners.

The level of statistical significance was set at *p* ≤ 0.05.

## 3. Results

A total of 2455 runners from D-A-CH countries (56% females and 44% males) constitutes the final sample included for statistical analysis. The median age was 37 (IQR 18, range: 18–74) years. According to the BMI categories, 83% (*n* = 2049) of participants had a normal weight, while the prevalence of underweight and overweight was 4% (*n* = 109) and 12% (*n* = 297), respectively. The majority of participants were from Germany (76%, *n* = 1866), followed by Austria with 431 participants (18%) and Switzerland with 158 participants (6%), respectively. The classification of runners based on race distance showed that 22% (*n* = 535) of participants were < 21-km runners, whereas 37% (*n* = 901) of participants were half-marathoners, and 42% (*n* = 1019) were (ultra-)marathoners. Runners reported to adhere to omnivorous (*n* = 1162), vegetarian (*n* = 529), and vegan (n = 764) diets by 47%, 22%, and 31%, respectively. In the present sample, the vegan diet among < 21-km runners (by 39%) and the omnivorous diet among half-marathoners (by 44%) and (ultra-)marathoners (by 56%) were more popular. 

Table 1 displays the participants’ sociodemographic characteristics across different dietary subgroups. A significant between-group difference was found for age, where omnivore runners had a higher mean age compared to vegetarians and vegans (*p* < 0.001). Distribution of females and males was significantly different among dietary groups, as females reported more often being vegetarians and vegans compared to males (*p* < 0.001). A significant difference between omnivores, vegetarians, and vegans was observed in BMI (*p* < 0.001), where the prevalence of underweight runners was lower in omnivores (2%) than vegetarians (6%) and vegans (6%), and contrariwise, overweight omnivores (16%) were more common than overweight vegetarians (8%) and vegans (9%). Comparison of race distance subgroups with dietary subgroups showed a significant difference between omnivores, vegetarians, and vegans (*p* < 0.001) (Table 1).

Analysis of individual records for completion of HM, M, and UM events showed that in female runners, the omnivores had a better average time for completion of HM (117.9 ± 18.5 min vs. 120.4 ± 19.0 min for vegetarians and 122.3 ± 18.8 min for vegans), and vegans in UM reported a better performance amongst dietary subgroups (748.2 ± 170.2 min vs. 776.5 ± 149.9 min for omnivores and 782.8 ± 206.5 min for vegetarians). In male runners, differences between dietary groups in the best time for completion of long-distance running events were not considerably remarkable, except for the poorest performance of vegetarian males in UM (758.8 ± 162.7 min vs. 708.0 ± 192.5 min for omnivores and 709.5 ± 162.4 min for vegans). Irrespective of diet type, data analysis showed that male half-marathoners, marathoners, and ultra-marathoners had faster finishing times for their running events with 16.8%, 15.4%, and 5.7%, respectively. Table 2 displays male and female runners’ best time to complete HM, M, and UM runs across different diet types. 

**Figure 2:**
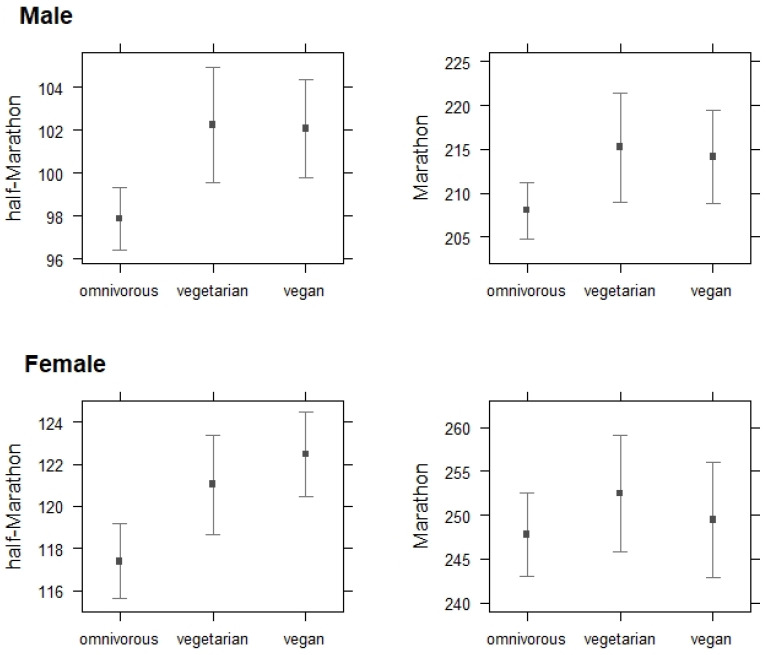
Effect plots with 95%-CI to show the differences between dietary subgroups in half-marathon and marathon performance in male and female recreational runners.

A linear regression model (explaining 11% of the variance over HM and 14% over M; *p* < 0.001) was fitted to find the marginal effect of diet type on female and male runners’ performances over HM and M distances. In males, compared to omnivores, vegans (+4.18 min; *p* < 0.001) and vegetarians (+4.38 min; *p* < 0.001) spent a longer time completing an HM; compared to omnivorous marathoners, vegetarian (but not vegan) marathoners spent 7.17 min (*p* < 0.05) longer to finish an M. In females, compared to omnivorous half-marathoners, vegans (+5.08 min; *p* < 0.001) and vegetarians (+3.65 min; *p* < 0.05) spent a longer time to complete an HM; there was no significant difference between dietary groups in M performance (*p* > 0.05). Figure 2 displays the 95% of confidence intervals to show differences between dietary subgroups in male and female average best runtimes over HM and M distances.

Table 3 shows the number of completed HM, M, and UM events in five categories. In total, 20% of half-marathoners, 27% of marathoners, and 25% of ultra-marathoners reported only one experience of completing a running event. While participation in at least one running event was required as an inclusion criterion, the maximum number of running participation reported was 76 events for half-marathon, 97 events for marathon, and 43 events for ultra-marathon. However, most runners in all three race distance subgroups reported less than seven completions of running events, and only 25% of half-marathoners, 23% of marathoners, and 16% of ultra-marathoners completed more than seven of the associated running events.

Analysis of training behaviors showed that 10% of the runners reported training and competing with supervision, and 91% of them reported following a professional trainer, while 12% and 10% had professional guidance by “sport scientists” and “sport medicine doctors,” respectively. Performance assessment was reported to be applied by 36% of runners, and consequently, 64% of runners did not assess their performance. Running frequency (i.e., number of running sessions) was found to be 3 days per week, with a range of 1–14 sessions. Regarding the training volume, runners reported training an average of 4.7 h per week, corresponding to 42.9 km of running. The participants’ daily and weekly training volume is shown in Table 4. 

**Figure 3:**
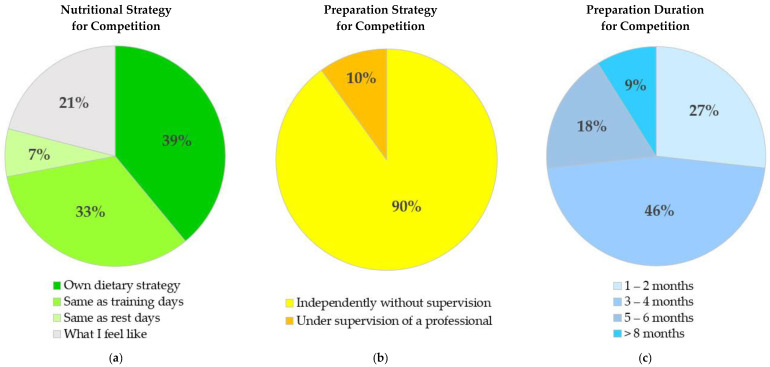
Pie charts demonstrating general strategies that endurance runners reported to prepare themselves for running competitions: (**a**) the prevalence of general nutritional strategies for running competitions based on four categories, with “own dietary strategy” as the most prevalent strategy; (**b**) the prevalence of general preparation strategies for running competitions based on two categories, with “independently without supervision” as the most prevalent strategy; (**c**) the prevalence of different durations for preparation of runners for running competitions based on four categories, with “3–4 months” as the most prevalent strategy.

General preparation strategies that runners reported to follow for running events are presented in Figure 3. Regarding nutritional approaches, 39% of runners reported following their own dietary strategy, and 33% reported continuing their training nutritional habits for races. The most prevalent duration that runners reported to prepare themselves for a running competition was “3–4 months” by 46%, followed by 1–2 months (27%), 5–6 months (18%), and >8 months (9%).

## 4. Discussion

Since scientific data about endurance athletes adhering to plant-based diets are limited, the NURMI Study (i.e., the largest running study in Europe) was designed to investigate a large number of participants with a valid data set in order to identify distinctions between dietary subgroups of recreational endurance runners. The objective of the NURMI Study Step 1 was to address the colloquial gross formulated question, “who is at the starting line of running events?” Therefore, the present study intended to investigate and compare the epidemiological characteristics (particularly diet types and running-associated behaviors) of active endurance runners in D-A-CH countries. The present study is the first and only study to scale the prevalence of omnivorous, vegetarian, and vegan runners at the starting line of running events.

The most important findings were that (i) there was a significant association between diet type and race distance; (ii) in females, vegan ultra-marathoners and omnivorous half-marathoners had better individual records in running (i.e., best time to complete their race distance) among dietary groups; (iii) sex differences in running performance had a minimizing trend with increasing race distance; (iv) the majority of runners (75% of half-marathoners, 77% of marathoners, and 84% of ultra-marathoners) reported to have completed less than seven running events; (v) most runners (90%) reported to prepare for running competitions independently over less than four months (73%); and (vi) runners reported an average distance of 42.9 ± 24.9 km (range: 5.0–187.5 km) regarding the weekly training volume. 

Decades of scientific investigation has been conducted to explicate the role of nutrition in endurance running; however, little is known regarding the nutritional data associated with diet types. According to the literature, both elite and recreational runners appear to suffer from nutrient deficiencies due to their athletic requirements [34,49]. Nutrient concerns might be more serious for distance runners who are known to be at a higher risk of low energy availability associated with their higher energy expenditure, particularly those who follow inappropriately planned diets [50]. While it has been documented that well-planned vegan/vegetarian diets can provide nutritional requirements of endurance activities [15], there are still some conflicts in popular beliefs around distance runners who follow a vegan/vegetarian diet [29]. Previous findings from our laboratory show that vegan distance runners have a higher consumption of dietary supplements to meet their micronutrient requirements [51] without differences between males and females [52]. Regardless of diet type, however, the patterns of supplement intake were similar between runners over different race distances [53]. In the present study, a positive association between diet type and race distance was observed, indicating the prevalence of vegan and vegetarian diets had an increasing trend with the advancement of the race distance. Consistently, other studies have also reported high prevalence rates of plant-based diets in ultra-marathoners compared to runners over shorter distances [29,54]. In the present study, the higher fraction of vegan and vegetarian participants could not be compared to similar studies on runners due to the sampling strategies implemented in the study design. However, research indicates that the prevalence of vegan/vegetarian diets among endurance runners is higher than among general populations [15,28,29,30]. The prevalence of vegan/vegetarian diets has also been reported to be associated with sociodemographic variables among endurance runners (e.g., sex, educational level, BMI, health status) [16,29,30] and general populations (e.g., sex, educational level, ethnicity) [26,46,55]. However, it should be considered that due to the restricted social environment in the past decades, people were formerly more reluctant to call themselves vegetarians or vegans, but nowadays, it is expressed as trendy to follow these diets and socially accepted as healthy [15,56]. The current COVID-19 pandemic has also increased the prevalence of plant-based diets [27], as it seems that plant-based diets could be a proper nutritional approach during the pandemic. In a study on 2884 front-line healthcare workers from different countries, findings showed that vegan/vegetarian participants had a 73% lower likelihood of a moderate-to-severe COVID-19 infection [57]. However, results from the NURMI Study Step 2 showed that vegetarian and vegan endurance runners had a high quality of life quite comparable with omnivorous runners, too [58]. Findings from our laboratory showed that vegan endurance runners contribute most beneficially to their overall state of health (range: 61–91% within eight dimensions). Conclusively, vegans reported to be more health-conscious than non-vegan runners, particularly due to the highest scores (75% ± 20%, *p* = 0.001) for their food-choice behavior. In line with laboratory reports on recreational runners [42,44,59], our previous results indicate that plant-based diets are compatible with endurance running [16].

In general, a major limitation in vegan/vegetarian prevalence studies is an undifferentiated and pooled analysis of vegetarian and vegan populations, which originates from the small percentage of vegans in studies with limited sample sizes. Therefore, caution is recommended when comparing the prevalence-related interpretations in the studies using the term vegetarian alone. Moreover, specific seasonal times could also affect the prevalence rates of vegan and vegetarian diets in some surveys, proposing that flexible vegan and vegetarian people may express different answers regarding their diet types in January than December (when many people make exceptions due to the festivities). In a large Swiss study [60], a survey regarding the prevalence of diet type has been conducted since 2015 (with around 8300 participants) to present (with more than 30,000 participants in 2020). Although the differentiation between vegan and vegetarians has only been considered since 2019, the continuous nature of this study provides viable information throughout the progression of time. Table 5 compares the prevalence of vegan and vegetarian diets in various European countries with a particular focus on the D-A-CH countries.

In the present study, a comparison of individual female records showed that omnivorous runners in HM and vegan runners in UM had a better running performance among dietary subgroups. However, no dietary-based differences in individual records were found in male runners, except for a poorer performance of vegetarian ultra-marathoners compared to their vegan and omnivorous counterparts. For the marginal effects considering diet type, the present findings show that compared to runners who follow a mixed diet, we found an increase in average time to complete HM distance by male vegetarians (4.38 min; *p* < 0.001) and vegans (4.18 min; *p* < 0.001) as well as female vegetarians (3.65 min; *p* < 0.05) and vegans (5.08 min; *p* < 0.001); however, the predominance of the omnivorous diet in HM performance was not observed in M performance in females. Although there are no comparable studies to equivalate these data to discuss and conclude the association between diet type and distance running performance, it seems that the level of professionalism (as a powerful modulator of performance) should be clearly controlled in interpretation of such data [30,69]. Results from an age-matched U.S.-based study on marathoners show that while the individual female records in M events are comparable to the present data (249 vs. 250 min), male marathoners in the present study had better individual records in M races (211 vs. 218 min), which might be partially associated with the nationality of the present sample from D-A-CH countries. In this regard, it is noteworthy that numerable ultra-endurance running events are held in D-A-CH countries, and most participants are part of the domestic populations [11]. An investigation of 150,710 UM finishers (between 1959 and 2016) has shown that runners from Japan, Germany, Switzerland, France, Italy, and the USA were more successful in completing the event [70]. In the present study, a sex-based comparison of individual records revealed that male half-marathoners, marathoners, and ultra-marathoners had faster times for completing their running events with 16.8%, 15.4%, and 5.7%, respectively. This trend suggests that sex-based differences in performance seem to disappear with increasing distance. While similar studies have consistently approved this sex-based outcome [71,72], the most reasonable explanations have been proposed for the sex-related differences in fuel utilization and muscle damage following ultra-endurance exercise [71].

In the pre-race preparation phase, a high training volume with long-distance runs has been associated with improved endurance and, consequently, a faster race time [73,74]. However, caution should be warranted regarding the negative consequences of overtraining, as it has been well-documented that a “faster and further” dosage fails from both health and performance viewpoints [35,75]. Runners in the present study reported having a weekly training volume of 42.9 km with a duration of 282 min and frequency of three sessions on average; the weekly exercise volume of runners was lower compared to recreational [28,34] and elite [36,76] marathoners from similar studies. Evidence indicates that the mean training volume of marathoners is about 50 km/week [34,36]. In general, race distance could be considered as an important indicator of training volume since runners over longer distances (e.g., marathoners and ultra-marathoners) were found to have greater training time and mileage compared to runners over shorter distances (e.g., half-marathoners) [37,38,41]. Regarding the training frequency, evidence shows that recreational runners complete an average of 3.7 runs per week, while elite runners complete 14.1 training runs per week [76]. According to the literature, there is a wide range of training volumes and frequencies reported by endurance runners [69,77], suggesting that a variety of modulating variables (not only limited to the professionalism level) is associated with the runners’ training volume.

The present findings show that 20–27% of runners over different race distances had only one experience of finishing a running event. On the other hand, however, 16–25% of participants were categorized as experienced runners with more than seven successful finishes. Although the average of completed runs is comparable to the findings from a similar study (~4 vs. ~4) [28], runners from the present study reported a wider range in completed events (HM: 1–76 runs, M: 1–97 runs, and UM: 1–43 runs) compared to the similar study conducted only on marathoners (1–10 runs) [28]. As an explanation, it should be considered that the opportunities for taking part in running events (which is high in D-A-CH countries, as mentioned earlier) and the motivations of recreational runners for participating in running events, particularly their health-related purposes, could potentially influence the number of races and finishing times [1]. Another determinant is the selection of specific races and seasons, which could affect the number of annual races, especially for recreational runners [41]. Evidence shows that recreational runners usually intend to take part in running events held throughout spring [41].

Pre-race preparation is considered the most important determining factor for endurance performance. Endurance runners may use different approaches to prepare themselves for running events [15]. Research suggests that being under the supervision of experts and professionals could bring about beneficial health- and performance-related advantages for endurance runners [39,40]. Data from the present study show that 9 in 10 runners train and prepare themselves for running competitions independently without supervision mostly (about 3 in 4) within less than four months. Among the runners who were under supervision, 91% reported training and competing under the supervision of a trainer, and only 12% and 10% of runners reported receiving supervision from “sport scientists” and “sport medicine doctors,” respectively. A prominent advantage of training supervision seems to be the assessment of adaptations and, accordingly, the replanning of training programs in a more purposeful procedure. However, only 36% of runners in the present study reported assessing their performance, which may be due to the high prevalence of health-related motives among recreational runners [6]. Evidence specifies that endurance runners who finish longer distances (i.e., marathon and ultra-marathon) receive more professional support in different phases of both racing and training compared to their counterparts in shorter distances (i.e., 10 km, HM) [42].

Similar to any investigation, this study has some limitations that should be mentioned. The NURMI Study was designed as cross-sectional with a self-report-based questionnaire approach, and thus, potential over- and under-reporting could affect the reliability of the data. However, using control questions implemented in different sections of the questionnaire, the likelihood of conflicting statements was minimized. Regarding the data clearance following control questions, we found an interesting result suggesting higher reliability of vegan participants compared to omnivores. In the process of data clearance, while 102 vegetarians were recategorized to omnivores, only five vegans incorrectly reported their category and were conclusively reclassified to the correct category. The higher proportion of vegan/vegetarian participants in the present study compared to D-A-CH countries and other parts of the world might be considered another important limitation affecting the interpretations. This result can be partly explained by the pre-selection of the participants, which might have affected the results; it was striking that most subjects came from Germany (76%), were females (56%), and followed a vegetarian/vegan diet (~53%), with the latter being markedly higher than the worldwide numbers and also D-A-CH countries (10–14%). Nonetheless, the high level of intrinsic motivation of the participants in this study may have led to an increase in their statement accuracy, thus contributing to a higher quality of the generated data. The imbalanced composition of our sample may be due to several reasons: (i) Germany, with a population of 82 million, is the largest German-speaking country [78] and (ii) has large vegetarian and vegan populations, too [79]. Regarding intrinsic motivations, however, it can be assumed that vegan and vegetarian runners might be more interested not only to participate in such lifestyle-approach studies but also to provide more detailed and reliable information because these investigations could be considered opportunities to express and expand their opinions without judgment and, vice versa, can consequently benefit results of their chosen lifestyles based on PA, sports, and exercise connected to plant-based diets. Hence, caution must be warranted in the representativeness of the findings. Additionally, variations among the respective running events, such as season, daytime, location of races, and environmental conditions (e.g., sun/rain, temperature, relative humidity), were uncontrolled variables that could potentially affect training and racing parameters. Therefore, the present investigation allows little conclusion regarding causality. However, this study provides valuable information and indicates who is at the start of a running event, especially being the first study with a specific focus on vegetarian and vegan runners, which is of particular interest for organizers of running events in general but also for trainers, coaches, and nutrition experts guiding athletes involved in running while adhering to some specific kind of diet. The present findings may help future investigations identify specific requirements of endurance runners when adhering to vegan and vegetarian diets.

## 5. Conclusions

The increasing popularity of participating in distance running events and the growing prevalence of vegan and vegetarian diets among endurance runners are two worldwide phenomena with a higher rate in D-A-CH countries. In the present study, in addition to the prevalence of 52.6% vegetarian/vegan and 47.4% omnivore runners at the start of events as well as discovering epidemiological findings associated with diet type and running parameters of recreational distance runners in D-A-CH countries, it was found that with increasing the race distance, there is a growing trend in the prevalence of vegan/vegetarian diet and simultaneously a decreasing trend in the sex-specific differences in runners’ records. These two findings are potentially connectable from an epidemiological viewpoint, suggesting a possible association of plant-based diets with the improvement of endurance performance in females. Conclusively, the present results indicate that vegetarian and vegan diets are compatible with competitive running. Findings from the present study provide a window into the targeted approaches of endurance athletes, running coaches/specialists, and sport scientists when applying training and nutritional strategies for endurance runners with different diet types. Future experimental studies can add support by conducting more detailed inquiries to provide specific data regarding the distinct association between endurance performance and different diet types among different sociodemographic categories of runners. 

## Figures and Tables

**Table 1:** Sociodemographic characteristics of participants presented by dietary subgroups.

	Total *n* = 2455	Omnivores *n* = 1162	Vegetarians *n* = 529	Vegans *n* = 764	Statistics
Age (years)	37 (IQR 18)	41 (IQR 17)	35 (IQR 17)	34 (IQR 15)	F_(2, 2452)_ = 47.77, *p* < 0.001
Weight (kg)	66 (IQR 16)	69 (IQR 16)	64 (IQR 14)	64 (IQR 15)	F_(2, 2452)_ = 40.44, *p* < 0.001
Height (m)	1.73 (IQR 0.13)	1.74 (IQR 0.12)	1.72 (IQR 0.12)	1.72 (IQR 0.13)	F_(2, 2452)_ = 11.23, *p* < 0.001
Sex					χ^2^_(2)_ = 72.16, *p* < 0.001
females	56% (1382)	47% (550)	64% (337)	65% (495)	
males	44% (1073)	53% (612)	36% (192)	35% (269)	
BMI (kg/m^2^)					χ^2^_(4)_ = 55.04, *p* < 0.001
<18.50	4% (109)	2% (29)	6% (32)	6% (48)	
18.50–24.99	83% (2049)	81% (942)	86% (457)	85% (650)	
>24.99	12% (297)	16% (191)	8% (40)	9% (66)	
Race distance					χ^2^_(4)_ = 60.93, *p* < 0.001
<21 km	22% (535)	17% (197)	24% (129)	27% (209)	
HM	37% (901)	34% (398)	37% (195)	40% (308)	
M/UM	42% (1019)	49% (567)	39% (205)	32% (247)	
Nationality					
Germany	76% (1866)	70% (816)	82% (434)	81% (616)	
Austria	18% (431)	20% (232)	14% (73)	16% (126)	
Switzerland	6% (158)	10% (114)	4% (22)	3% (22)	

Data are presented as median (along with IQR) or prevalence (along with number of participants). BMI BMI—body mass index. χ^2^—chi-square. F—F-test. *p*—*p*-value for difference among groups. <21 KM—distances shorter than half-marathon. HM—half-marathon. M/UM—marathon/ultra-marathon. Omnivores—those who have no restriction on source of food. Vegetarians—those who avoid all flesh foods but consume egg and/or dairy products. Vegans—those who avoid all foods and ingredients from animal sources.

**Table 2:** Best time as individual records for completion of half-marathon, marathon, and ultra-marathon events in male and female runners based on dietary subgroups.

	Female Runners *n* = 1382			Male Runners *n* = 1073		
	Omnivores *n* = 550	Vegetarians *n* = 337	Vegans *n* = 495	Omnivores *n* = 612	Vegetarians *n* = 192	Vegans *n* = 269
HM (minutes)	117.9 ± 18.5 (51.0–235.0)	120.4 ± 19.0 (74.0–213.4)	122.3 ± 18.8 (61.3–215.6)	99.0 ± 16.8 (58.4–235.0)	101.0 ± 17.1 (70.2–189.2)	100.2 ± 17.8 (44.0–167.0)
M (minutes)	249.5 ± 34.9 (126.2–365.1)	249.7 ± 35.5 (173.6–342.1)	248.9 ± 39.6 (169.8–428.0)	210.2 ± 33.4 (141.7–360.4)	212.4 ± 33.4 (149.6–310.8)	210.4 ± 28.2 (145.6–293.0)
UM (minutes)	776.5 ± 149.9 (576.0–1,102.9)	782.8 ± 206.5 (547.0–1,185.2)	748.2 ± 170.2 (438.8–1,071.7)	708.0 ± 192.5 (363.9–1,599.1)	758.8 ± 162.7 (487.2–1,117.8)	709.5 ± 162.4 (483.1–1,198.8)

Data are presented as mean ± standard deviation (inclusive range). Omnivores —those who have no restriction on source of food. Vegetarians—those who avoid all flesh foods but consume egg and/or dairy products. Vegans—those who avoid all foods and ingredients from animal sources. HM—half-marathon. M—marathon. UM—ultra-marathon.

**Table 3:** Number of half-marathon, marathon, and ultra-marathon competitions completed by runners (as successful participation in the running events) based on numerical categories.

	Range	1 Time	2 Times	3–4 Times	5–7 Times	>7 Times	Not Reported
HM (*n* = 1712)	1–76 events	20% (341)	18% (316)	21% (359)	16% (273)	25% (420)	<1% (3)
M (*n* = 972)	1–97 events	27% (259)	18% (178)	19% (187)	13% (123)	23% (221)	<1% (4)
UM (*n* = 232)	1–43 events	25% (59)	19% (45)	23% (54)	16% (37)	16% (37)	-

Data are presented as percentage (along with number of participants). HM—half-marathon; M—marathon; UM—ultra-marathon.

**Table 4:** Training volume of recreational endurance runners (*n* = 2455).

	Daily		Weekly	
	Mean ± SD	Range	Mean ± SD	Range
Training Duration (h)	0.85 ± 0.47	0.22–7.75	4.7 ± 2.7	0.6–20.5
Training Distance (km)	13.1 ± 7.3	3.4–120.0	42.9 ± 24.9	5.0–187.5

SD—standard deviation. h—hours. km—kilometers.

**Table 5:** The prevalence of vegan/vegetarian diets in D-A-CH countries and two other German-associated countries compared to Europe.

		Vegetarian	Vegan	Reference
Germany (D)	2016	4.3% *		[26]
	2018	6.0%	1.0%	[25]
	2019	6.0%	1.0%	[61]
	2020	5.0%	1.0%	[62]
	2021	10.0%	2.0%	[25]
Austria (A)	2018	8.3%	1.2%	[63]
Switzerland (CH)	2015	2.9% *		[60]
	2017	3.2% *		
	2018	3.5% *		
	2019	3.8%	0.2%	
	2020	3.7%	0.3%	
	2021	4.7%	0.6%	
Belgium	2016	2.0%	0.3%	[64]
	2018	1.7%	0.5%	
	2020	2.4%	0.6%	
Italy	2015	5.9%*		[65,66,67]
	2016	8.0%*		
	2017	7.6%*		
	2018	6.2%	0.9%	
	2019	5.4%	1.9%	
	2020	6.7%	2.2%	
	2021	5.8%	2.4%	
Europe	2020	7.0%	2.0%	[68]

* Pooled results of vegan and vegetarian categories. D-A-CH—Germany, Austria, and Switzerland.

## Data Availability

The data sets generated during and/or analyzed during the current study are not publicly available but may be made available upon reasonable request. Subjects will receive a brief summary of the results of the NURMI Study if desired.
